# Structure-Based and Rational Design of a Hepatitis C Virus Vaccine

**DOI:** 10.3390/v13050837

**Published:** 2021-05-05

**Authors:** Johnathan D. Guest, Brian G. Pierce

**Affiliations:** 1 University of Maryland Institute for Bioscience and Biotechnology Research, Rockville, MD 20850, USA; jdguest@terpmail.umd.edu; 2 Department of Cell Biology and Molecular Genetics, University of Maryland, College Park, MD 20742, USA

**Keywords:** HCV, E1E2, structure-based vaccine design

## Abstract

A hepatitis C virus (HCV) vaccine is a critical yet unfulfilled step in addressing the global disease burden of HCV. While decades of research have led to numerous clinical and pre-clinical vaccine candidates, these efforts have been hindered by factors including HCV antigenic variability and immune evasion. Structure-based and rational vaccine design approaches have capitalized on insights regarding the immune response to HCV and the structures of antibody-bound envelope glycoproteins. Despite successes with other viruses, designing an immunogen based on HCV glycoproteins that can elicit broadly protective immunity against HCV infection is an ongoing challenge. Here, we describe HCV vaccine design approaches where immunogens were selected and optimized through analysis of available structures, identification of conserved epitopes targeted by neutralizing antibodies, or both. Several designs have elicited immune responses against HCV in vivo, revealing correlates of HCV antigen immunogenicity and breadth of induced responses. Recent studies have elucidated the functional, dynamic and immunological features of key regions of the viral envelope glycoproteins, which can inform next-generation immunogen design efforts. These insights and design strategies represent promising pathways to HCV vaccine development, which can be further informed by successful immunogen designs generated for other viruses.

## 1. Introduction

Hepatitis C virus (HCV) represents a global disease burden, with approximately 71 million people infected [1]. The majority of untreated HCV infections become chronic [2,3] and may lead to cirrhosis or hepatocellular carcinoma (HCC), a deadly type of liver cancer [4,5]. Although direct-acting antiviral (DAA) drugs have cure rates greater than 90% [6,7], they do not prevent a recurrence of HCV infection [8] and may not reduce the risk of HCC [9,10]. Combined with financial barriers and the asymptomatic nature of many HCV infections [11,12], treatment with DAAs alone has not been enough to stop HCV transmission, and development of an effective vaccine for HCV is still viewed as essential [13,14]. However, efforts to produce an HCV vaccine, many of which have been described in previous reviews [15,16,17,18,19], have thus far been unsuccessful. Multiple factors likely contribute to the difficulty in developing an HCV vaccine [20,21], including substantial diversity between genotypes [22,23], viral mutation in infected individuals leading to quasispecies that can escape neutralizing antibodies [24], epitope shielding by glycans on the E1 and E2 envelope proteins [25,26], epitope shielding by apolipoproteins in HCV lipo-viral-particles (LVPs) [27,28,29], and other mechanisms of immune evasion [30,31]. Current limitations of and lack of standardization for in vitro and in vivo models of HCV infection may also hinder the evaluation and comparison of vaccine candidates [13,32]. Additionally, a high-resolution structure of the E1E2 glycoprotein complex, which is the target of neutralizing antibodies against HCV and thought to be a trimer of heterodimers on the surface of the virion [33], has not yet been determined, due in part to structural flexibility [34] and the requirement of hydrophobic transmembrane domains for assembly [35,36]. Structural characterization of envelope glycoprotein assemblies for other viruses has been facilitated by a trimerization domain as a scaffold [37,38], a modified furin cleavage site [39], or targeted stabilizing mutations [40,41,42], enabling structure-based vaccine designs for those antigens [43,44]. Remarkable progress was achieved even in human immunodeficiency virus (HIV) despite challenges of diversity, flexibility, and glycan shielding in the Env glycoproteins [45,46,47] that are broadly similar to challenges observed for HCV and E1E2.

Though the structure of the E1E2 heterodimer is not known, broadly neutralizing antibody (bnAb) interactions with E1 and E2 have been structurally characterized, providing insights into the neutralization determinants of known epitopes that may be crucial for stimulating protective B cell responses [48,49]. Conserved clusters of epitopes on E2 have been classified either as antigenic domains A-E (nomenclature used for this review) [50,51,52], epitopes I–III [20], or antigenic regions (ARs) 1–3 [53], and the AR classification also includes E1E2 epitopes (AR4, AR5) [54]. Although different epitope clusters can overlap [31,55], epitope mapping and structural studies have identified the following key E2 regions for bnAb recognition: antigenic domain B (residues 529–535 in H77 isolate numbering), domain D (residues 434–446), and domain E (residues 412–423), all of which contain residues critical for antibody binding that are nearly or fully conserved across genotypes [56,57]. Antibodies targeting these three antigenic domains of E2 neutralize the virus through competition with CD81, an HCV co-receptor that is critical for viral entry [58,59,60]. Conserved epitopes targeted by bnAbs have also been mapped to E1 (residues 314–324) [61] and the E1E2 heterodimer; the latter epitopes collectively include hotspot residues in both the stem region of E2 and the N-terminus of E1 [49,54,62]. On the other hand, antigenic domain A, which has been mapped to a region that includes E2 (residues 627–637) [31], is considered unnecessary for eliciting bnAbs, as antibodies that target this epitope are non-neutralizing [63].

Structure-based HCV vaccine design can be guided by this information, as sequence and structural determinants for many bnAb-associated epitopes are well known [55,56,64], with the notable exceptions of bnAb epitopes on the E1E2 heterodimer and the structural context of E1 and E2 epitopes that would be provided by the E1E2 ectodomain structure. However, large gaps in the structural knowledge of E1E2 have led to designs of the heterodimer that increase stability and solubility while maintaining or improving immunogenicity. In this review, we summarize recent structure-based and rational vaccine design efforts that harness current structural and antigenic knowledge of HCV glycoproteins to generate improved immunogens and, in some cases, to help elucidate the ectodomain structure of E1E2. The path to an effective HCV vaccine may include utilization of design strategies that generated promising immunogens for other viruses, including HIV, RSV, and SARS-CoV-2 [38,39,40,41,42], which have led to clinical-stage candidates [65,66,67] and successful vaccines [68,69].

## 2. Design Approaches

### 2.1. Epitope Scaffolding and Epitope-Based Designs

**Figure 1:**
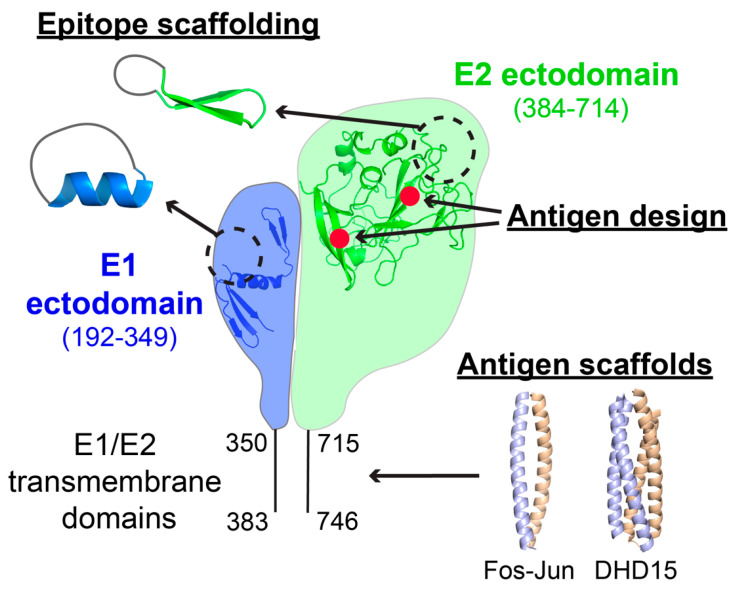
Structure-based approaches for HCV E1E2 vaccine design. Approaches shown represent: (1) scaffolding of epitopes from E1 and E2, (2) design of the E2 antigen through truncation or residue substitutions to alter antigenicity, immunogenicity, or epitope exposure, and (3) scaffolding of E1E2 to generate stable, secreted glycoproteins. These design strategies are labeled as “Epitope scaffolding”, “Antigen design”, and “Antigen scaffolds”, respectively. Molecular structures shown are from PDB codes 4N0Y (E1 epitope) [61], 5KZP (E2 epitope) [77], 4UOI (E1 N-terminal ectodomain) [79], 6MEI (E2 ectodomain core) [80], 1FOS (Fos-Jun E1E2 scaffold) [81] and 6DMA (DHD15 E1E2 scaffold) [82], and were rendered in PyMOL v. 1.8 (Schrödinger, LLC). Red points on E2 represent rationally selected modifications of E2 (residue substitutions or loop truncations). Residue ranges for ectodomains and transmembrane domains are labeled according to H77 numbering.

Numerous structure-based HCV vaccine design efforts have focused on utilizing conserved glycoprotein epitopes as immunogens and stabilizing one or more of these epitopes, either through scaffolding or cyclization. These strategies attempt to focus responses to key epitopes and have been employed for RSV and HIV immunogen designs to elicit more neutralizing antibodies than the unmodified proteins [38,70]. This design approach, along with others described in this review, is shown in Figure 1. Targets of epitope-based designs have included domain E (also referred to as Epitope I or AS412), a highly conserved linear epitope near the N-terminus of E2 that has been structurally characterized in complex with multiple bnAbs [71,72,73,74]. In many cases, scaffolded and epitope-based designs have been tested for immunogenicity and elicitation of neutralizing antibodies in vivo; these studies are described along with other in vivo-tested HCV immunogen designs in Table 1. 

Informed by the β-hairpin conformation of the domain E epitope found in multiple bnAb-bound structures [71,72], Sandomenico et al. designed a cyclized version named C-Epitope I, with E2 residues 411 and 423 mutated to cysteines that form a disulfide bridge to present epitope residues 412–422 [75]. This cyclized peptide was injected into mice following conjugation to a carrier protein, which was either keyhole limpet hemocyanin (KLH) or bovine serum albumin (BSA). Seven monoclonal antibodies that recognized C-Epitope I were isolated from murine hybridomas and showed pronounced binding specificity for the cyclic peptide design. Some of these isolated antibodies bound to the native epitope in the context of E2 with nanomolar affinity, but were found to be non-neutralizing. The structure of C-Epitope I was determined in complex with one of the induced murine monoclonal antibodies, then compared to the linear peptide bound to bnAb AP33 [71]. This comparison suggested that the conformationally-restrained C-Epitope I may not recapitulate the native β-hairpin, in part because a domain E residue that is typically glycosylated (N417) [25] was partially buried in the cyclized peptide interface with the monoclonal antibody [75]. 

A similar study generated designs of cyclized domain E peptides, but instead utilized the Protein Data Bank (PDB) [76] to compare the β-hairpin conformation with other protein structures in order to find a template for a cyclized peptide that would mimic the bnAb-bound epitope [77]. These designed immunogens bound to the neutralizing antibody HCV1 [72], and a crystal structure of HCV1 in complex with one cyclized peptide, named C1, confirmed that the native β-hairpin conformation and key bnAb interactions were retained as designed. Serum neutralization levels induced by these designs were relatively limited, but the cyclic peptides were still found to be immunogenic and induced antibodies that neutralized H77 HCV pseudoparticles (HCVpp), representing an improvement in immunogenicity over the linear (non-cyclic) peptide control [77]. Aside from cyclization modes that were different from the C-Epitope I design reported by Sandomenico et al., it is worth noting that the domain E peptides used in the Pierce et al. study included an N-glycan at position N417 (reflective of the glycan in native E2), which may have helped to induce the detectable, but low, neutralizing antibody responses in mice. Another study searched for structures that could provide a scaffold for either the domain E epitope or an E1 epitope, which adopts a helical conformation when bound to bnAb IGH526 [61,78]. For each epitope, the bnAb-bound conformations were compared to a large database of structures from the PDB, with structural similarities evaluated by six alignment algorithms [78]. Following this selection process, each target epitope was grafted onto a structurally similar scaffold and optimized, effectively presenting these HCV epitopes in a new context. Designs of scaffolded domain E and IGH526 epitopes were recognized by bnAbs known to target the native epitopes, and their antigenicity was increased through multimeric display of select scaffolded designs with self-assembling ferritin nanoparticles [78].

Several other studies have reported and characterized epitope-based HCV vaccine designs that do not utilize structure-based design or scaffolding of the epitopes themselves. Recently, Cowton et al. generated and structurally characterized an anti-idiotype monoclonal antibody, named B2.1A, that was recognized by AP33, effectively designing a structural mimic of domain E in a β-hairpin conformation [83]. Antibodies induced by B2.1A protected against HCV challenge from genotype 1 or 2 strains in an immunosuppressed and humanized liver chimeric mouse model, suggesting that B2.1A could form the basis of distinct immunogen designs focused on antigenic domain E [83]. The development of synthetic peptide libraries derived from HCV E1E2 found several peptides similar to hypervariable region 1 (HVR1) [84], domain E, and domain D that exhibited robust antigenicity in ELISA and elicited neutralizing murine antibodies in vivo when linked to a T-helper cell epitope [85,86]. Another peptide vaccine that combined the epitope from IGH526-bound E1, epitopes from E2 domains C and D, and peptides from nonstructural HCV proteins showed stimulation of humoral and cellular immunity in mice [87]. Cyclic peptides that are recognized by domain D antibodies have been generated through immobilization by synthetic conjugation, effectively mimicking a discontinuous and conformational epitope [88]. As reviewed by others [89], multiple studies have capitalized on self-assembly of hepatitis B virus (HBV) capsid proteins [90] to insert epitopes from domain E [91,92], HVR1 [93,94,95], and additional E2 antigenic domains [96] into exposed loops of the HBV capsid protein. These designs formed chimeric and stable virus-like particles (VLPs), and a few designs elicited immune responses with some capacity to neutralize multiple genotypes [91,96].

**Table 1:** E2 and E1E2 immunogen designs tested in vivo.

Design(s)	In Vivo Data	Viruses Tested for Neutralization ^1^	Results ^2^	Reference
Cyclized domain E peptides, truncated E2 core with additional domain E and HVR1 removed	Mouse	Homologous HCVpp: H77 Heterologous HCVpp: genotypes 1b, 4a	Neutralized H77 in HCVpp assays	[77]
E2 core with proline mutations in domain D or E, glycosylation of domain A, HVR1 removed	Mouse	Homologous HCVpp: H77 Heterologous HCVpp: genotypes 1b, 2a, 2i, 4a	Increased neutralization of heterologous strains in HCVpp assays	[97]
E2 core with truncated and designed HVR2, displayed on nanoparticles	Mouse	Homologous HCVpp: H77 Heterologous HCVpp: genotypes 1a, 2a, 5a	Increased neutralization of H77 in HCVpp assays	[98]
Cyclized and conjugated domain E peptide	Mouse	Homologous HCVcc: H77 Heterologous viruses: not tested	Antibody isolated from mice non-neutralizing in HCVcc assays	[75]
E2 core with eight cysteines mutated	Mouse	Homologous HCVpp: H77 Heterologous viruses: not tested	No neutralization of H77 in HCVpp assays	[99]
E1E2 ectodomains oligomerized with C4b-binding protein IMX313P	Mouse	Homologous virus: not tested Heterologous HCVpp: genotypes 1a, 1b, 2a, 2b, 3a, 4, 5, 6	Neutralized most strains in HCVpp assays	[100]
E2 core with HVR1 and HVR2 removed, IgVR deglycosylated, and displayed on nanoparticles	Mouse	Homologous HCVpp: H77 Heterologous HCVpp: genotype 1b	Increased neutralization of H77 in HCVpp assays	[101]
E2 core fused to ferritin	Mouse	Homologous HCVcc: Con1 Heterologous HCVcc: genotypes 1a, 1b, 2a, 3a, 4a, 5a, 7a	Increased neutralization of strains in HCVcc assays	[102]
Synthetic consensus of E2 core from genotype 1 sequences	Guinea pig	Homologous HCVpp: NotC1 Heterologous HCVpp: genotypes 1a, 1b, 2a, 3	Increased neutralization of H77 in HCVpp assays	[103]
E2 core with variable regions removed and seven cysteines mutated	Guinea pig	Homologous HCVpp: H77 Heterologous HCVcc: genotypes 2a, 3a, 5a	Neutralized H77 HCVpp, limited neutralization of HCVcc	[104]
Combination of two HVR1 peptides	Mouse	Homologous HCVpp: C47 Heterologous HCVpp: genotypes 1a, 1b, 2a, 3a, 4a, 5a, 6a	Neutralized strains in HCVpp assays	[105]
E2 with mutated N-glycan sites and HVR1 removed	Mouse	Homologous HCVcc: Jc1 Heterologous HCVcc: genotype 5a Heterologous HCVpp: genotype 1a	Neutralized Jc1 HCVcc, H77 HCVpp	[106]
E2 epitopes from domains B, D, and E, and HVR1 mimotope displayed on HBV-S VLPs	Mouse	Homologous virus: not tested Heterologous HCVpp and HCVcc: genotypes 1a, 1b, 2a	Neutralized strains in HCVpp and HCVcc assays	[91]
E2 epitopes from domains B-E displayed on HBV-S VLPs	Mouse	Homologous virus: not tested Heterologous HCVcc: genotypes 1a, 1b, 2a, 2b, 3a, 4a, 5a, 6a	IgGs purified from sera neutralized some strains in HCVcc assays	[96]
E1E2 ectodomains with C-terminal leucine zipper as scaffold, furin cleavage site	Mouse	Homologous HCVpp: H77 Heterologous viruses: not tested	Neutralized H77 in HCVpp assays	[107]

^1^ Homologous virus is listed by strain name; heterologous viruses are listed by genotype and subtype when known. ^2^ Summary of measured neutralization induced by design, or improvement in neutralization over non-designed control, if measured by the authors. Unless otherwise noted, results refer to serum neutralization.

### 2.2. E2 Antigen Design

Though the E2 ectodomain core (residues 384–661; also defined as residues 384–645 [99]) can be expressed as an immunogen alone [77,108,109], its wild-type form may not be optimal for generating bnAb responses, due to possible factors noted earlier such as occlusion of bnAb epitopes with glycans, glycoprotein dynamics, and the presence of non-neutralizing epitopes. Insights regarding the structural basis of bnAb recognition provide an opportunity to modify specific epitopes in the context of E2 to improve immunogenicity. The aforementioned Pierce et al. study identified structural similarity between the β-hairpin conformation of domain E and a region of domain A, which as noted above is associated with non-neutralizing antibodies, and replaced the domain A residues with a copy of the domain E epitope in two E2 core designs, but found no significant differences in neutralization potency compared to an unmodified E2 core when tested for immunogenicity in mice [77]. Another study engineered E2 to improve immunogenicity by modifying domain A and D epitopes [97]. First, a proline mutation was introduced at residue 445 in domain D to stabilize the epitope in a conformation recognized by domain D antibody HC84.26.WH.5DL [97,110]. After another analysis of E2 core structure, an N-glycosylation site was added at residue 632, masking the domain A site to decrease the elicitation of non-neutralizing antibodies targeting that region [97]. Though the proline substitution increased HC84.26.WH.5DL binding affinity and the N-glycan addition greatly reduced domain A antibody binding, only the proline substitution led to both increased neutralization of multiple HCVpp representing neutralization-resistant HCV strains and maintained neutralization of homologous and other heterologous HCVpp [97]. Additional research has focused on optimizing the E2 core to increase antigenicity and stability. Optimized E2 cores were designed using sequences derived from genotype 1 or genotype 6 strains [98] and were structurally characterized in complex with multiple bnAbs [80,111,112]. These optimized cores improved on previous constructs [80,111] by truncating and designing flexible HVR2 and β-sandwich loops, preserving E2 core antigenicity while increasing thermostability [98]. Top designs of E2 core were also displayed on ferritin and other nanoparticle assemblies, exhibiting improved immunogenicity [98]. Other studies have tested multivalent presentation of E2 core on nanoparticles. Display of E2 core on lipid-based vesicles [101] or ferritin [102] has led to higher neutralization of HCVpp or HCV cell-cultured virus (HCVcc) [113] and stronger binding to neutralizing antibodies than E2 core alone, demonstrating the benefit of multimeric display for optimizing immunogenicity. E2 core aggregation through disulfide cross-linking was addressed in another design, where removal of eight cysteine residues resulted in the production of monomeric E2 core [99]. However, the monomeric E2 core with cysteines removed did not stimulate T cell responses as well as the wild type, aggregated E2 core, and no groups of mice immunized with the design or other constructs showed detectable viral neutralization [99].

Other efforts to improve E2 antigenicity and immunogenicity have optimized sequences and removed flexible variable regions, offering a complement to structure-based designs. Based on sequence analysis, primarily of genotype 1a, synthetic consensus sequences of E2 have been reported, but have not consistently improved immunogenicity [103,114]. HVR1 has been removed in many E2 core designs [77,101,104,115], as it can interfere with and enable evasion of bnAb responses [116,117]. In one case, HVR1 was found to determine sensitivity to heterologous neutralization by sera from goats immunized with an E1E2 vaccine candidate, in part through differential binding with co-receptor SR-BI [118]. Along with other variable regions, HVR1 was removed in an E2 core design that maintained binding to CD81 and formed an apparent homodimer [119,120]. A high-molecular-weight form of this E2 core design showed promising immunogenicity in guinea pigs [121], and has been further optimized through mutation of cysteine residues [104]. However, removing HVR1 alone does not appear to improve the immunogenicity of vaccine designs [115,122]. Removal of HVR1 also had minimal effects on antigenicity and immunogenicity when combined with mutations on E2 core that either removed glycans or modulated recognition of antigenic domains A and D [97,106]. Counterintuitively, a bivalent peptide vaccine designed from HVR1 has induced neutralization of heterologous HCV strains [105].

### 2.3. E1E2 Optimization

While some design efforts have focused on presenting specific neutralizing epitopes, other groups have pursued larger and more complex scaffolds to optimize native-like assembly of E1E2 proteins, often by removing or replacing hydrophobic transmembrane domains [123] to produce a stable and soluble vaccine candidate. Cao et al. designed and characterized E1E2 constructs with either a C-terminal Fc-tag or a designed heterodimeric scaffold to enforce heterodimerization of soluble E1E2 and enable structural and functional characterization of E1E2, which has been hindered by the difficulties in producing that complex in homogenous form [124]. These constructs were expressed in either insect or mammalian cells and were identified as a mixture of heterodimeric and higher-order oligomers. Structure-based design of a soluble E1E2 heterodimer provided an opportunity to characterize this assembly with unprecedented detail. Along with evidence of heterogeneity, Cao et al. demonstrated binding of several neutralizing antibodies to both E1E2 constructs, though the antibodies were limited to those that target E2 domains B or E, or an E1 epitope. Expression of stable E1E2 constructs also allowed for low-resolution electron microscopy reconstructions of E1E2 with the designed heterodimeric scaffold, which were compared to a computational prediction of the E1E2 glycoprotein structure generated using E1E2 sequence co-evolution [124]. Another study reported the design of several constructs that replaced E1E2 transmembrane domains with scaffolds, in conjunction with a furin cleavage site to permit expression of the designs as a cleaved polyprotein, identifying a design with a human leucine zipper scaffold (sE1E2.LZ) that displayed native-like antigenicity and immunogenicity [107]. Though this construct was somewhat heterogeneous based on analytical characterization, it was shown to be smaller and less complex than full-length E1E2 that was extracted from the cell membrane with intact transmembrane domains. Antigenic characterization confirmed that sE1E2.LZ bound with high affinity not only to antibodies that target E2 antigenic domains A-E and E1E2, but also to CD81. Neutralization of homologous (H77) HCVpp by sE1E2.LZ-immunized murine sera was comparable to neutralization from sera immunized with full-length E1E2, suggesting that this scaffolded E1E2 represents a secreted and native-like form of the E1E2 heterodimer [107].

Additional efforts have designed E1E2 constructs for optimal secretion and have tested for native-like properties with antibody binding assays. One study described several designs of secreted E1E2 that removed E1 and E2 transmembrane domains and connected the ectodomains with various tags and linkers, including one design with a cleavage site between E1 and E2 [125]. These designs showed binding to multiple antibodies targeting different epitopes, but an sE1E2 construct in a DNA format induced only limited serum neutralization in mice [125]. Another effort expressed and purified E1E2 with the C-terminal oligomerization domain of C4b-binding protein, resulting in E1E2 heptamers that elicited immune responses from mice with some capacity to neutralize HCVpp [100].

### 2.4. New and Alternative Approaches

While a number of rational E2, E1E2, and epitope-based designs have been tested, recent studies have yielded new structural and mechanistic insights regarding HCV immune evasion and entry that may inform prospective designs. Notably, the impact of conformational flexibility in E2 regions, especially HVR1 and domain E, on antibody resistance and receptor binding has been explored [34,126,127]. In comparing resistant sequences with the sensitive reference sequence H77 [128], mutations in a five-residue motif in HVR1 were found to directly contribute to differences in neutralization resistance, corroborating previous findings [129]. These HVR1 variants were more dependent on the SR-BI co-receptor for entry, and showed a higher propensity to adopt β-hairpin conformations in domain E. In addition, SR-BI dependency was strongly correlated with accessibility of the CD81-binding site and other bnAb epitopes, suggesting that CD81 may induce a conformational transition from closed to open in a subsequent attachment step that is dependent on the presence of HVR1 [122,130]. Closed and open states of E2 were linked to distinct conformations of domain E through temperature-dependent neutralization assays, with a β-hairpin associated with a closed state, and an extended, HC33.1 bnAb-bound epitope conformation associated with an open state [126]. With an improved mechanistic understanding of E2 conformational dynamics, structure-based vaccine designs can better account for the effect of inherent flexibility on bnAb epitope accessibility and immune evasion.

A variety of vaccines have been designed for HCV without using E1E2, instead utilizing antigens from core [131,132,133], p7 [134] or non-structural proteins [135,136], which have been evaluated for stimulation of T cell responses [137,138], with a focus on DNA vaccines [139,140,141] or expression with viral vectors [142,143]. One T cell-based vaccine with viral vectored non-structural proteins has been tested in a phase 1/2 clinical trial for protection against HCV infection in healthy at-risk individuals; while it failed to prevent chronic HCV infection, it did induce HCV-specific T cell responses [144]. Rationally designed T cell antigens, possibly in conjunction with B cell antigens, may be a means to improve efficacy of such approaches. Vaccine designs combining the HCV core protein with E1E2 can induce both B cell and T cell responses, as shown by a VLP assembly of four different HCV genotypes [145] and a chimeric protein with epitopes from all three antigens and NS3 [146]. Although p7 has not been the focus of vaccine studies until recently, overlapping peptides of the antigen displayed on nanoparticles stimulated significantly higher T cell responses in mice, providing another avenue for vaccine development [147]. Rational design approaches have also been utilized to stimulate CD8^+^ T cell responses by accounting for HCV genetic diversity. Two studies created synthetic genotype 1 sequences through mosaic and ancestral sequence methods [114,148], and were validated for in vivo immunogenicity or T cell stimulation [149,150]. Of note, the mosaic vaccine approach was previously utilized for HIV antigen design [151], and mosaic HIV antigens were found to be immunogenic in a phase 1/2a clinical trial (NCT02315703) and protective against simian-human immunodeficiency virus (SHIV) infection in rhesus macaques [152].

## 3. Discussion

The strategies of glycoprotein optimization, design, and scaffolding have produced a variety of rational and structure-based designs of HCV vaccine candidates. Collectively, these approaches have provided useful data and insights into determinants of immunogenicity and bnAb elicitation, as well as novel platforms for epitope and glycoprotein presentation. Despite this progress, the ability to conduct rational HCV vaccine design would dramatically increase with greater structural information on E1E2 glycoproteins, as demonstrated by immunogens generated using structure-based design for influenza [153,154,155], HIV [39,156], and SARS-CoV-2 [42,69,157,158], which have known glycoprotein complex structures available for reference in design studies. High-resolution structural characterization of the E1E2 heterodimer would be immensely useful in this regard, while useful insights would also be gained through the structure of E2 bound to CD81, and any component of the complex interactions between HCV LVPs and multiple lipoprotein receptors [29]. As an alternative to structural characterization of secreted, soluble E1E2 ectodomains, characterization of the native, membrane-bound E1E2 heterodimer can be investigated using methods such as cryogenic electron microscopy with lipid nanodiscs, which has already been used for characterization of full-length HIV glycoprotein assemblies [159]. 

In future studies, designed HCV antigens can utilize approaches such as nanoparticle or multivalent display of E1E2 or key epitopes, in conjunction with T cell antigen designs for other HCV proteins, to optimize immunogenicity while effectively coordinating stimulation of broad B cell and T cell responses. With multivalent HCV antigens showing promising results in at least two studies [105,145], incorporating multivalency may be advantageous for future candidates given the high sequence variability of HCV and past success with other viruses, specifically regarding the elicitation of immune responses and protection from challenge in animal models [160]. Finally, complete and consistent evaluations of vaccine designs may be impractical without overcoming current limitations in animal models and assessments of immunogenicity, including the lack of available immunocompetent animal models for HCV challenge studies, and the use of murine and guinea pig models in immunogenicity studies, as these species are unable to reflect key features of human bnAb responses to HCV [80,161] due to differences in antibody germline genes. Several of these limitations have been addressed by recent work, including the finding that E1E2 immunization in rhesus macaques can generate bnAbs with sequence and structural features that are similar to human bnAbs [162,163], and the identification and use of hepaciviruses as HCV surrogates for immunocompetent in vivo studies [164,165]. The many recent advances in HCV immunogen design and vaccine assessment, along with lessons and concepts from vaccine design efforts for other viruses and pathogens, can enable the successful development of an optimal and effective HCV vaccine.

## Data Availability

Data discussed in this review are available in the cited publications, and structural coordinates shown in Figure 1 are from the Protein Data Bank (https://www.rcsb.org (accessed on 26 April 2021)).

## References

[1] WHO (2017). Global Hepatitis Report 2017.

[2] Alberti A., Chemello L., Benvegnu L. (1999). Natural history of hepatitis C. J. Hepatol..

[3] Micallef J.M., Kaldor J.M., Dore G.J. (2006). Spontaneous Viral. clearance following acute hepatitis C infection: A systematic review of longitudinal studies. J. Viral. Hepat..

[4] Sebastiani G., Gkouvatsos K., Pantopoulos K. (2014). Chronic hepatitis C and liver fibrosis. World J. Gastroenterol..

[5] Forner A., Reig M., Bruix J. (2018). Hepatocellular carcinoma. Lancet.

[6] Hayes C.N., Chayama K. (2017). Why highly effective drugs are not enough: The need for an affordable solution to eliminating HCV. Expert Rev. Clin. Pharmacol..

[7] Baumert T.F., Berg T., Lim J.K., Nelson D.R. (2019). Status of Direct-Acting AntiViral. Therapy for Hepatitis C Virus Infection and Remaining Challenges. Gastroenterology.

[8] Bartenschlager R., Baumert T.F., Bukh J., Houghton M., Lemon S.M., Lindenbach B.D., Lohmann V., Moradpour D., Pietschmann T., Rice C.M. (2018). Critical challenges and emerging opportunities in hepatitis C virus research in an era of potent antiViral. therapy: Considerations for scientists and funding agencies. Virus Res..

[9] Roche B., Coilly A., Duclos-Vallee J.C., Samuel D. (2018). The impact of treatment of hepatitis C with DAAs on the occurrence of HCC. Liver Int..

[10] Guarino M., Sessa A., Cossiga V., Morando F., Caporaso N., Morisco F., Special Interest Group on “Hepatocellular carcinoma and new anti-HCV therapies” of the Italian Association for the Study of the Liver (2018). Direct-acting antivirals and hepatocellular carcinoma in chronic hepatitis C: A few lights and many shadows. World J. Gastroenterol..

[11] Hajarizadeh B., Grebely J., Dore G.J. (2013). Epidemiology and natural history of HCV infection. Nat. Rev. Gastroenterol. Hepatol..

[12] Calvaruso V., Petta S., Craxi A. (2018). Is global elimination of HCV realistic?. Liver Int.

[13] Duncan J.D., Urbanowicz R.A., Tarr A.W., Ball J.K. (2020). Hepatitis C Virus Vaccine: Challenges and Prospects. Vaccines.

[14] Walker C.M., Grakoui A. (2015). Hepatitis C virus: Why do we need a vaccine to prevent a curable persistent infection?. Curr. Opin. Immunol..

[15] Honegger J.R., Zhou Y., Walker C.M. (2014). Will there be a vaccine to prevent HCV infection?. Semin. Liver Dis..

[16] Bailey J.R., Barnes E., Cox A.L. (2019). Approaches, Progress, and Challenges to Hepatitis C Vaccine Development. Gastroenterology.

[17] Fauvelle C., Colpitts C.C., Keck Z.Y., Pierce B.G., Foung S.K., Baumert T.F. (2016). Hepatitis C virus vaccine candidates inducing protective neutralizing antibodies. Expert Rev. Vaccines.

[18] Sepulveda-Crespo D., Resino S., Martinez I. (2020). Hepatitis C virus vaccine design: Focus on the humoral immune response. J. Biomed. Sci..

[19] Freeman Z.T., Cox A.L. (2016). Lessons from Nature: Understanding Immunity to HCV to Guide Vaccine Design. PLoS Pathog..

[20] Drummer H.E. (2014). Challenges to the development of vaccines to hepatitis C virus that elicit neutralizing antibodies. Front. Microbiol..

[21] Fuerst T.R., Pierce B.G., Keck Z.Y., Foung S.K.H. (2017). Designing a B Cell-Based Vaccine against a Highly Variable Hepatitis C Virus. Front. Microbiol..

[22] Tarr A.W., Khera T., Hueging K., Sheldon J., Steinmann E., Pietschmann T., Brown R.J. (2015). Genetic Diversity Underlying the Envelope Glycoproteins of Hepatitis C Virus: Structural and Functional Consequences and the Implications for Vaccine Design. Viruses.

[23] Smith D.B., Bukh J., Kuiken C., Muerhoff A.S., Rice C.M., Stapleton J.T., Simmonds P. (2014). Expanded classification of hepatitis C virus into 7 genotypes and 67 subtypes: Updated criteria and genotype assignment web resource. Hepatology.

[24] Babcock G.J., Iyer S., Smith H.L., Wang Y., Rowley K., Ambrosino D.M., Zamore P.D., Pierce B.G., Molrine D.C., Weng Z. (2014). High-throughput sequencing analysis of post-liver transplantation HCV E2 glycoprotein evolution in the presence and absence of neutralizing monoclonal antibody. PLoS ONE.

[25] Lavie M., Hanoulle X., Dubuisson J. (2018). Glycan Shielding and Modulation of Hepatitis C Virus Neutralizing Antibodies. Front. Immunol..

[26] Helle F., Duverlie G., Dubuisson J. (2011). The hepatitis C virus glycan shield and evasion of the humoral immune response. Viruses.

[27] Wrensch F., Crouchet E., Ligat G., Zeisel M.B., Keck Z.Y., Foung S.K.H., Schuster C., Baumert T.F. (2018). Hepatitis C Virus (HCV)-Apolipoprotein Interactions and Immune Evasion and Their Impact on HCV Vaccine Design. Front. Immunol..

[28] Di Lorenzo C., Angus A.G., Patel A.H. (2011). Hepatitis C virus evasion mechanisms from neutralizing antibodies. Viruses.

[29] Cosset F.L., Mialon C., Boson B., Granier C., Denolly S. (2020). HCV Interplay with Lipoproteins: Inside or Outside the Cells?. Viruses.

[30] Pantua H., Diao J., Ultsch M., Hazen M., Mathieu M., McCutcheon K., Takeda K., Date S., Cheung T.K., Phung Q. (2013). Glycan shifting on hepatitis C virus (HCV) E2 glycoprotein is a mechanism for escape from broadly neutralizing antibodies. J. Mol. Biol..

[31] Pierce B.G., Keck Z.Y., Foung S.K. (2016). Viral. evasion and challenges of hepatitis C virus vaccine development. Curr. Opin. Virol..

[32] Riva L., Dubuisson J. (2019). Similarities and Differences Between HCV Pseudoparticle (HCVpp) and Cell Culture HCV (HCVcc) in the Study of HCV. Methods Mol. Biol..

[33] Falson P., Bartosch B., Alsaleh K., Tews B.A., Loquet A., Ciczora Y., Riva L., Montigny C., Montpellier C., Duverlie G. (2015). Hepatitis C Virus Envelope Glycoprotein E1 Forms Trimers at the Surface of the Virion. J. Virol..

[34] Stejskal L., Lees W.D., Moss D.S., Palor M., Bingham R.J., Shepherd A.J., Grove J. (2020). Flexibility and intrinsic disorder are conserved features of hepatitis C virus E2 glycoprotein. PLoS Comput. Biol..

[35] Ciczora Y., Callens N., Penin F., Pecheur E.I., Dubuisson J. (2007). Transmembrane domains of hepatitis C virus envelope glycoproteins: Residues involved in E1E2 heterodimerization and involvement of these domains in virus entry. J. Virol..

[36] Op De Beeck A., Montserret R., Duvet S., Cocquerel L., Cacan R., Barberot B., Le Maire M., Penin F., Dubuisson J. (2000). The transmembrane domains of hepatitis C virus envelope glycoproteins E1 and E2 play a major role in heterodimerization. J. Biol. Chem..

[37] Walls A.C., Park Y.J., Tortorici M.A., Wall A., McGuire A.T., Veesler D. (2020). Structure, Function, and Antigenicity of the SARS-CoV-2 Spike Glycoprotein. Cell.

[38] McLellan J.S., Chen M., Joyce M.G., Sastry M., Stewart-Jones G.B., Yang Y., Zhang B., Chen L., Srivatsan S., Zheng A. (2013). Structure-based design of a fusion glycoprotein vaccine for respiratory syncytial virus. Science.

[39] Sanders R.W., Derking R., Cupo A., Julien J.P., Yasmeen A., de Val N., Kim H.J., Blattner C., de la Pena A.T., Korzun J. (2013). A next-generation cleaved, soluble HIV-1 Env trimer, BG505 SOSIP.664 gp140, expresses multiple epitopes for broadly neutralizing but not non-neutralizing antibodies. PLoS Pathog..

[40] Julien J.P., Cupo A., Sok D., Stanfield R.L., Lyumkis D., Deller M.C., Klasse P.J., Burton D.R., Sanders R.W., Moore J.P. (2013). Crystal structure of a soluble cleaved HIV-1 envelope trimer. Science.

[41] Pallesen J., Wang N., Corbett K.S., Wrapp D., Kirchdoerfer R.N., Turner H.L., Cottrell C.A., Becker M.M., Wang L., Shi W. (2017). Immunogenicity and structures of a rationally designed prefusion MERS-CoV spike antigen. Proc. Natl. Acad. Sci. USA.

[42] Hsieh C.L., Goldsmith J.A., Schaub J.M., DiVenere A.M., Kuo H.C., Javanmardi K., Le K.C., Wrapp D., Lee A.G., Liu Y. (2020). Structure-based design of prefusion-stabilized SARS-CoV-2 spikes. Science.

[43] Graham B.S., Gilman M.S.A., McLellan J.S. (2019). Structure-Based Vaccine Antigen Design. Annu. Rev. Med..

[44] Sesterhenn F., Bonet J., Correia B.E. (2018). Structure-based immunogen design-leading the way to the new age of precision vaccines. Curr. Opin. Struct. Biol..

[45] Wagh K., Hahn B.H., Korber B. (2020). Hitting the sweet spot: Exploiting HIV-1 glycan shield for induction of broadly neutralizing antibodies. Curr. Opin. HIV AIDS.

[46] Munro J.B., Lee K.K. (2018). Probing Structural Variation and Dynamics in the HIV-1 Env Fusion Glycoprotein. Curr. HIV Res..

[47] Bbosa N., Kaleebu P., Ssemwanga D. (2019). HIV subtype diversity worldwide. Curr. Opin. HIV AIDS.

[48] Guest J.D., Pierce B.G. (2018). Computational Modeling of Hepatitis C Virus Envelope Glycoprotein Structure and Recognition. Front. Immunol..

[49] Colbert M.D., Flyak A.I., Ogega C.O., Kinchen V.J., Massaccesi G., Hernandez M., Davidson E., Doranz B.J., Cox A.L., Crowe J.E. (2019). Broadly Neutralizing Antibodies Targeting New Sites of Vulnerability in Hepatitis C Virus E1E2. J. Virol..

[50] Keck Z.Y., Li T.K., Xia J., Bartosch B., Cosset F.L., Dubuisson J., Foung S.K. (2005). Analysis of a highly flexible conformational immunogenic domain a in hepatitis C virus E2. J. Virol..

[51] Keck Z.Y., Xia J., Wang Y., Wang W., Krey T., Prentoe J., Carlsen T., Li A.Y., Patel A.H., Lemon S.M. (2012). Human monoclonal antibodies to a novel cluster of conformational epitopes on HCV E2 with resistance to neutralization escape in a genotype 2a isolate. PLoS Pathog..

[52] Keck Z., Wang W., Wang Y., Lau P., Carlsen T.H., Prentoe J., Xia J., Patel A.H., Bukh J., Foung S.K. (2013). Cooperativity in virus neutralization by human monoclonal antibodies to two adjacent regions located at the amino terminus of hepatitis C virus E2 glycoprotein. J. Virol..

[53] Law M., Maruyama T., Lewis J., Giang E., Tarr A.W., Stamataki Z., Gastaminza P., Chisari F.V., Jones I.M., Fox R.I. (2008). Broadly neutralizing antibodies protect against hepatitis C virus quasispecies challenge. Nat. Med..

[54] Giang E., Dorner M., Prentoe J.C., Dreux M., Evans M.J., Bukh J., Rice C.M., Ploss A., Burton D.R., Law M. (2012). Human broadly neutralizing antibodies to the envelope glycoprotein complex of hepatitis C virus. Proc. Natl. Acad. Sci. USA.

[55] Pierce B.G., Keck Z.Y., Lau P., Fauvelle C., Gowthaman R., Baumert T.F., Fuerst T.R., Mariuzza R.A., Foung S.K.H. (2016). Global mapping of antibody recognition of the hepatitis C virus E2 glycoprotein: Implications for vaccine design. Proc. Natl. Acad. Sci. USA.

[56] Stroh L.J., Krey T. (2020). HCV Glycoprotein Structure and Implications for B-Cell Vaccine Development. Int. J. Mol. Sci..

[57] Mancini N., Diotti R.A., Perotti M., Sautto G., Clementi N., Nitti G., Patel A.H., Ball J.K., Clementi M., Burioni R. (2009). Hepatitis C virus (HCV) infection may elicit neutralizing antibodies targeting epitopes conserved in all Viral. genotypes. PLoS ONE.

[58] Stroh L.J., Nagarathinam K., Krey T. (2018). Conformational Flexibility in the CD81-Binding Site of the Hepatitis C Virus Glycoprotein E2. Front. Immunol..

[59] Bose M., Mullick R., Das S., Das S., Karande A.A. (2016). Combination of neutralizing monoclonal antibodies against Hepatitis C virus E2 protein effectively blocks virus infection. Virus Res..

[60] Feneant L., Levy S., Cocquerel L. (2014). CD81 and hepatitis C virus (HCV) infection. Viruses.

[61] Kong L., Kadam R.U., Giang E., Ruwona T.B., Nieusma T., Culhane J.C., Stanfield R.L., Dawson P.E., Wilson I.A., Law M. (2015). Structure of Hepatitis C Virus Envelope Glycoprotein E1 Antigenic Site 314-324 in Complex with Antibody IGH526. J. Mol. Biol..

[62] Bailey J.R., Wasilewski L.N., Snider A.E., El-Diwany R., Osburn W.O., Keck Z., Foung S.K., Ray S.C. (2015). Naturally selected hepatitis C virus polymorphisms confer broad neutralizing antibody resistance. J. Clin. Investig..

[63] Keck Z.Y., Xia J., Cai Z., Li T.K., Owsianka A.M., Patel A.H., Luo G., Foung S.K. (2007). Immunogenic and functional organization of hepatitis C virus (HCV) glycoprotein E2 on infectious HCV virions. J. Virol..

[64] Gopal R., Jackson K., Tzarum N., Kong L., Ettenger A., Guest J., Pfaff J.M., Barnes T., Honda A., Giang E. (2017). Probing the antigenicity of hepatitis C virus envelope glycoprotein complex by high-throughput mutagenesis. PLoS Pathog..

[65] Evaluating the Safety and Immunogenicity of HIV-1 BG505 SOSIP.664 gp140 With TLR Agonist and/or Alum Adjuvants in Healthy, HIV-Uninfected Adults. https://ClinicalTrials.gov/show/NCT04177355.

[66] Ruckwardt T.J., Morabito K.M., Phung E., Crank M.C., Costner P.J., Holman L.A., Chang L.A., Hickman S.P., Berkowitz N.M., Gordon I.J. (2021). Safety, tolerability, and immunogenicity of the respiratory syncytial virus prefusion F subunit vaccine DS-Cav1: A phase 1, randomised, open-label, dose-escalation clinical trial. Lancet Respir. Med..

[67] Dose, Safety, Tolerability and Immunogenicity of an Influenza H1 Stabilized Stem Ferritin Vaccine, VRCFLUNPF099-00-VP, in Healthy Adults. https://ClinicalTrials.gov/show/NCT03814720.

[68] Baden L.R., El Sahly H.M., Essink B., Kotloff K., Frey S., Novak R., Diemert D., Spector S.A., Rouphael N., Creech C.B. (2021). Efficacy and Safety of the mRNA-1273 SARS-CoV-2 Vaccine. N. Engl. J. Med..

[69] Corbett K.S., Edwards D.K., Leist S.R., Abiona O.M., Boyoglu-Barnum S., Gillespie R.A., Himansu S., Schafer A., Ziwawo C.T., DiPiazza A.T. (2020). SARS-CoV-2 mRNA vaccine design enabled by prototype pathogen preparedness. Nature.

[70] Hessell A.J., Powell R., Jiang X., Luo C., Weiss S., Dussupt V., Itri V., Fox A., Shapiro M.B., Pandey S. (2019). Multimeric Epitope-Scaffold HIV Vaccines Target V1V2 and Differentially Tune Polyfunctional Antibody Responses. Cell Rep..

[71] Potter J.A., Owsianka A.M., Jeffery N., Matthews D.J., Keck Z.Y., Lau P., Foung S.K., Taylor G.L., Patel A.H. (2012). Toward a Hepatitis C Virus Vaccine: The Structural Basis of Hepatitis C Virus Neutralization by AP33, a Broadly Neutralizing Antibody. J. Virol..

[72] Kong L., Giang E., Robbins J.B., Stanfield R.L., Burton D.R., Wilson I.A., Law M. (2012). Structural basis of hepatitis C virus neutralization by broadly neutralizing antibody HCV1. Proc. Natl. Acad. Sci. USA.

[73] Meola A., Tarr A.W., England P., Meredith L.W., McClure C.P., Foung S.K., McKeating J.A., Ball J.K., Rey F.A., Krey T. (2015). Structural flexibility of a conserved antigenic region in hepatitis C virus glycoprotein E2 recognized by broadly neutralizing antibodies. J. Virol..

[74] Li Y., Pierce B.G., Wang Q., Keck Z.Y., Fuerst T.R., Foung S.K., Mariuzza R.A. (2015). Structural basis for penetration of the glycan shield of hepatitis C virus E2 glycoprotein by a broadly neutralizing human antibody. J. Biol. Chem..

[75] Sandomenico A., Leonardi A., Berisio R., Sanguigno L., Foca G., Foca A., Ruggiero A., Doti N., Muscariello L., Barone D. (2016). Generation and Characterization of Monoclonal Antibodies against a Cyclic Variant of Hepatitis C Virus E2 Epitope 412-422. J. Virol..

[76] Berman H.M., Westbrook J., Feng Z., Gilliland G., Bhat T.N., Weissig H., Shindyalov I.N., Bourne P.E. (2000). The Protein Data Bank. Nucleic Acids Res..

[77] Pierce B.G., Boucher E.N., Piepenbrink K.H., Ejemel M., Rapp C.A., Thomas W.D., Sundberg E.J., Weng Z., Wang Y. (2017). Structure-Based Design of Hepatitis C Virus Vaccines That Elicit Neutralizing Antibody Responses to a Conserved Epitope. J. Virol..

[78] He L., Cheng Y., Kong L., Azadnia P., Giang E., Kim J., Wood M.R., Wilson I.A., Law M., Zhu J. (2015). Approaching rational epitope vaccine design for hepatitis C virus with meta-server and multivalent scaffolding. Sci. Rep..

[79] El Omari K., Iourin O., Kadlec J., Sutton G., Harlos K., Grimes J.M., Stuart D.I. (2014). Unexpected structure for the N-terminal domain of hepatitis C virus envelope glycoprotein E1. Nat. Commun..

[80] Flyak A.I., Ruiz S., Colbert M.D., Luong T., Crowe J.E., Bailey J.R., Bjorkman P.J. (2018). HCV Broadly Neutralizing Antibodies Use a CDRH3 Disulfide Motif to Recognize an E2 Glycoprotein Site that Can Be Targeted for Vaccine Design. Cell Host Microbe.

[81] Glover J.N., Harrison S.C. (1995). Crystal structure of the heterodimeric bZIP transcription factor c-Fos-c-Jun bound to DNA. Nature.

[82] Chen Z., Boyken S.E., Jia M., Busch F., Flores-Solis D., Bick M.J., Lu P., VanAernum Z.L., Sahasrabuddhe A., Langan R.A. (2019). Programmable design of orthogonal protein heterodimers. Nature.

[83] Cowton V.M., Owsianka A.M., Fadda V., Ortega-Prieto A.M., Cole S.J., Potter J.A., Skelton J.K., Jeffrey N., Di Lorenzo C., Dorner M. (2021). Development of a structural epitope mimic: An idiotypic approach to HCV vaccine design. NPJ Vaccines.

[84] Prentoe J., Bukh J. (2018). Hypervariable Region 1 in Envelope Protein 2 of Hepatitis C Virus: A Linchpin in Neutralizing Antibody Evasion and Viral. Entry. Front. Immunol..

[85] Grollo L., Torresi J., Drummer H., Zeng W., Williamson N., Jackson D.C. (2006). Exploiting information inherent in binding sites of virus-specific antibodies: Design of an HCV vaccine candidate cross-reactive with multiple genotypes. Antivir. Ther..

[86] Torresi J., Stock O.M., Fischer A.E., Grollo L., Drummer H., Boo I., Zeng W., Earnest-Silveira L., Jackson D.C. (2007). A self-adjuvanting multiepitope immunogen that induces a broadly cross-reactive antibody to hepatitis C virus. Hepatology.

[87] Dawood R.M., Moustafa R.I., Abdelhafez T.H., El-Shenawy R., El-Abd Y., Bader El Din N.G., Dubuisson J., El Awady M.K. (2019). A multiepitope peptide vaccine against HCV stimulates neutralizing humoral and persistent cellular responses in mice. BMC Infect. Dis..

[88] Meuleman T.J., Dunlop J.I., Owsianka A.M., van de Langemheen H., Patel A.H., Liskamp R.M.J. (2018). Immobilization by Surface Conjugation of Cyclic Peptides for Effective Mimicry of the HCV-Envelope E2 Protein as a Strategy toward Synthetic Vaccines. Bioconjug. Chem..

[89] Torresi J. (2017). The Rationale for a Preventative HCV Virus-Like Particle (VLP) Vaccine. Front. Microbiol..

[90] Hu J., Liu K. (2017). Complete and Incomplete Hepatitis B Virus Particles: Formation, Function, and Application. Viruses.

[91] Wei S., Lei Y., Yang J., Wang X., Shu F., Wei X., Lin F., Li B., Cui Y., Zhang H. (2018). Neutralization effects of antibody elicited by chimeric HBV S antigen viral-like particles presenting HCV neutralization epitopes. Vaccine.

[92] Czarnota A., Tyborowska J., Peszynska-Sularz G., Gromadzka B., Bienkowska-Szewczyk K., Grzyb K. (2016). Immunogenicity of Leishmania-derived hepatitis B small surface antigen particles exposing highly conserved E2 epitope of hepatitis C virus. Microb. Cell Fact..

[93] Netter H.J., Macnaughton T.B., Woo W.P., Tindle R., Gowans E.J. (2001). Antigenicity and immunogenicity of novel chimeric hepatitis B surface antigen particles with exposed hepatitis C virus epitopes. J. Virol..

[94] Netter H.J., Woo W.P., Tindle R., Macfarlan R.I., Gowans E.J. (2003). Immunogenicity of recombinant HBsAg/HCV particles in mice pre-immunised with hepatitis B virus-specific vaccine. Vaccine.

[95] Vietheer P.T., Boo I., Drummer H.E., Netter H.J. (2007). Immunizations with chimeric hepatitis B virus-like particles to induce potential anti-hepatitis C virus neutralizing antibodies. Antivir. Ther..

[96] Czarnota A., Offersgaard A., Pihl A.F., Prentoe J., Bukh J., Gottwein J.M., Bienkowska-Szewczyk K., Grzyb K. (2020). Specific Antibodies Induced by Immunization with Hepatitis B Virus-Like Particles Carrying Hepatitis C Virus Envelope Glycoprotein 2 Epitopes Show Differential Neutralization Efficiency. Vaccines.

[97] Pierce B.G., Keck Z.Y., Wang R., Lau P., Garagusi K., Elkholy K., Toth E.A., Urbanowicz R.A., Guest J.D., Agnihotri P. (2020). Structure-Based Design of Hepatitis C Virus E2 Glycoprotein Improves Serum Binding and Cross-Neutralization. J. Virol..

[98] He L., Tzarum N., Lin X., Shapero B., Sou C., Mann C.J., Stano A., Zhang L., Nagy K., Giang E. (2020). Proof of concept for rational design of hepatitis C virus E2 core nanoparticle vaccines. Sci. Adv..

[99] Marin M.Q., Sliepen K., Garcia-Arriaza J., Koekkoek S.M., Perez P., Sorzano C.O.S., Gomez C.E., Sanders R.W., Esteban M. (2020). Optimized Hepatitis C Virus (HCV) E2 Glycoproteins and their Immunogenicity in Combination with MVA-HCV. Vaccines.

[100] Masavuli M.G., Wijesundara D.K., Underwood A., Christiansen D., Earnest-Silveira L., Bull R., Torresi J., Gowans E.J., Grubor-Bauk B. (2019). A Hepatitis C Virus DNA Vaccine Encoding a Secreted, Oligomerized Form of Envelope Proteins Is Highly Immunogenic and Elicits Neutralizing Antibodies in Vaccinated Mice. Front. Immunol..

[101] Bazzill J.D., Ochyl L.J., Giang E., Castillo S., Law M., Moon J.J. (2018). Interrogation of Antigen Display on Individual Vaccine Nanoparticles for Achieving Neutralizing Antibody Responses against Hepatitis C Virus. Nano Lett..

[102] Yan Y., Wang X., Lou P., Hu Z., Qu P., Li D., Li Q., Xu Y., Niu J., He Y. (2020). A Nanoparticle-Based Hepatitis C Virus Vaccine With Enhanced Potency. J. Infect. Dis..

[103] Tarr A.W., Backx M., Hamed M.R., Urbanowicz R.A., McClure C.P., Brown R.J.P., Ball J.K. (2018). Immunization with a synthetic consensus hepatitis C virus E2 glycoprotein ectodomain elicits virus-neutralizing antibodies. Antivir. Res..

[104] Center R.J., Boo I., Phu L., McGregor J., Poumbourios P., Drummer H.E. (2020). Enhancing the antigenicity and immunogenicity of monomeric forms of hepatitis C virus E2 for use as a preventive vaccine. J. Biol. Chem..

[105] Mosa A.I., Urbanowicz R.A., AbouHaidar M.G., Tavis J.E., Ball J.K., Feld J.J. (2020). A bivalent HCV peptide vaccine elicits pan-genotypic neutralizing antibodies in mice. Vaccine.

[106] Khera T., Behrendt P., Bankwitz D., Brown R.J.P., Todt D., Doepke M., Khan A.G., Schulze K., Law J., Logan M. (2019). Functional and immunogenic characterization of diverse HCV glycoprotein E2 variants. J. Hepatol..

[107] Guest J.D., Wang R., Elkholy K.H., Chagas A., Chao K.L., Cleveland T.E.t., Kim Y.C., Keck Z.Y., Marin A., Yunus A.S. (2021). Design of a native-like secreted form of the hepatitis C virus E1E2 heterodimer. Proc. Natl. Acad. Sci. USA.

[108] Alhammad Y., Gu J., Boo I., Harrison D., McCaffrey K., Vietheer P.T., Edwards S., Quinn C., Coulibaly F., Poumbourios P. (2015). Monoclonal Antibodies Directed toward the Hepatitis C Virus Glycoprotein E2 Detect Antigenic Differences Modulated by the N-Terminal Hypervariable Region 1 (HVR1), HVR2, and Intergenotypic Variable Region. J. Virol..

[109] Broering T.J., Garrity K.A., Boatright N.K., Sloan S.E., Sandor F., Thomas W.D., Szabo G., Finberg R.W., Ambrosino D.M., Babcock G.J. (2009). Identification and characterization of broadly neutralizing human monoclonal antibodies directed against the E2 envelope glycoprotein of hepatitis C virus. J. Virol..

[110] Keck Z.Y., Wang Y., Lau P., Lund G., Rangarajan S., Fauvelle C., Liao G.C., Holtsberg F.W., Warfield K.L., Aman M.J. (2016). Affinity maturation of a broadly neutralizing human monoclonal antibody that prevents acute hepatitis C virus infection in mice. Hepatology.

[111] Kong L., Giang E., Nieusma T., Kadam R.U., Cogburn K.E., Hua Y., Dai X., Stanfield R.L., Burton D.R., Ward A.B. (2013). Hepatitis C virus E2 envelope glycoprotein core structure. Science.

[112] Tzarum N., Giang E., Kong L., He L., Prentoe J., Augestad E., Hua Y., Castillo S., Lauer G.M., Bukh J. (2019). Genetic and structural insights into broad neutralization of hepatitis C virus by human VH1-69 antibodies. Sci. Adv..

[113] Steinmann E., Pietschmann T. (2013). Cell culture systems for hepatitis C virus. Curr. Top. Microbiol. Immunol..

[114] Munshaw S., Bailey J.R., Liu L., Osburn W.O., Burke K.P., Cox A.L., Ray S.C. (2012). Computational reconstruction of Bole1a, a representative synthetic hepatitis C virus subtype 1a genome. J. Virol..

[115] Law J.L.M., Logan M., Wong J., Kundu J., Hockman D., Landi A., Chen C., Crawford K., Wininger M., Johnson J. (2018). Role of the E2 Hypervariable Region (HVR1) in the Immunogenicity of a Recombinant Hepatitis C Virus Vaccine. J. Virol..

[116] Keck Z.Y., Girard-Blanc C., Wang W., Lau P., Zuiani A., Rey F.A., Krey T., Diamond M.S., Foung S.K. (2016). Antibody Response to Hypervariable Region 1 Interferes with Broadly Neutralizing Antibodies to Hepatitis C Virus. J. Virol..

[117] Prentoe J., Verhoye L., Velazquez Moctezuma R., Buysschaert C., Farhoudi A., Wang R., Alter H., Meuleman P., Bukh J. (2015). HVR1-mediated antibody evasion of highly infectious in vivo adapted HCV in humanised mice. Gut.

[118] Johnson J., Freedman H., Logan M., Wong J.A.J., Hockman D., Chen C., He J., Beard M.R., Eyre N.S., Baumert T.F. (2019). A Recombinant Hepatitis C Virus Genotype 1a E1/E2 Envelope Glycoprotein Vaccine Elicits Antibodies That Differentially Neutralize Closely Related 2a Strains through Interactions of the N-Terminal Hypervariable Region 1 of E2 with Scavenger Receptor B1. J. Virol..

[119] McCaffrey K., Boo I., Poumbourios P., Drummer H.E. (2007). Expression and characterization of a minimal hepatitis C virus glycoprotein E2 core domain that retains CD81 binding. J. Virol..

[120] McCaffrey K., Boo I., Owczarek C.M., Hardy M.P., Perugini M.A., Fabri L., Scotney P., Poumbourios P., Drummer H.E. (2017). An Optimized Hepatitis C Virus E2 Glycoprotein Core Adopts a Functional Homodimer That Efficiently Blocks Virus Entry. J. Virol..

[121] Vietheer P.T., Boo I., Gu J., McCaffrey K., Edwards S., Owczarek C., Hardy M.P., Fabri L., Center R.J., Poumbourios P. (2017). The core domain of hepatitis C virus glycoprotein E2 generates potent cross-neutralizing antibodies in guinea pigs. Hepatology.

[122] Prentoe J., Velazquez-Moctezuma R., Augestad E.H., Galli A., Wang R., Law M., Alter H., Bukh J. (2019). Hypervariable region 1 and N-linked glycans of hepatitis C regulate virion neutralization by modulating envelope conformations. Proc. Natl. Acad. Sci. USA.

[123] Lavie M., Goffard A., Dubuisson J. (2007). Assembly of a functional HCV glycoprotein heterodimer. Curr. Issues Mol. Biol..

[124] Cao L., Yu B., Kong D., Cong Q., Yu T., Chen Z., Hu Z., Chang H., Zhong J., Baker D. (2019). Functional expression and characterization of the envelope glycoprotein E1E2 heterodimer of hepatitis C virus. PLoS Pathog..

[125] Ruwona T.B., Giang E., Nieusma T., Law M. (2014). Fine mapping of murine antibody responses to immunization with a novel soluble form of hepatitis C virus envelope glycoprotein complex. J. Virol..

[126] Augestad E.H., Castelli M., Clementi N., Stroh L.J., Krey T., Burioni R., Mancini N., Bukh J., Prentoe J. (2020). Global and local envelope protein dynamics of hepatitis C virus determine broad antibody sensitivity. Sci. Adv..

[127] Balasco N., Barone D., Iaccarino E., Sandomenico A., De Simone A., Ruvo M., Vitagliano L. (2018). Intrinsic structural versatility of the highly conserved 412-423 epitope of the Hepatitis C Virus E2 protein. Int. J. Biol. Macromol..

[128] von Hahn T., Yoon J.C., Alter H., Rice C.M., Rehermann B., Balfe P., McKeating J.A. (2007). Hepatitis C virus continuously escapes from neutralizing antibody and T-cell responses during chronic infection in vivo. Gastroenterology.

[129] El-Diwany R., Cohen V.J., Mankowski M.C., Wasilewski L.N., Brady J.K., Snider A.E., Osburn W.O., Murrell B., Ray S.C., Bailey J.R. (2017). Extra-epitopic hepatitis C virus polymorphisms confer resistance to broadly neutralizing antibodies by modulating binding to scavenger receptor B1. PLoS Pathog..

[130] Prentoe J., Jensen T.B., Meuleman P., Serre S.B., Scheel T.K., Leroux-Roels G., Gottwein J.M., Bukh J. (2011). Hypervariable region 1 differentially impacts viability of hepatitis C virus strains of genotypes 1 to 6 and impairs virus neutralization. J. Virol..

[131] Gawlik K., Gallay P.A. (2014). HCV core protein and virus assembly: What we know without structures. Immunol. Res..

[132] Roohvand F., Aghasadeghi M.R., Sadat S.M., Budkowska A., Khabiri A.R. (2007). HCV core protein immunization with Montanide/CpG elicits strong Th1/Th2 and long-lived CTL responses. Biochem. Biophys. Res. Commun..

[133] Drane D., Maraskovsky E., Gibson R., Mitchell S., Barnden M., Moskwa A., Shaw D., Gervase B., Coates S., Houghton M. (2009). Priming of CD4+ and CD8+ T cell responses using a HCV core ISCOMATRIX vaccine: A phase I study in healthy volunteers. Hum. Vaccin.

[134] Madan V., Bartenschlager R. (2015). Structural and Functional Properties of the Hepatitis C Virus p7 Viroporin. Viruses.

[135] Thimme R. (2021). T cell immunity to hepatitis C virus: Lessons for a prophylactic vaccine. J. Hepatol..

[136] Mekonnen Z.A., Grubor-Bauk B., Masavuli M.G., Shrestha A.C., Ranasinghe C., Bull R.A., Lloyd A.R., Gowans E.J., Wijesundara D.K. (2019). Toward DNA-Based T-Cell Mediated Vaccines to Target HIV-1 and Hepatitis C Virus: Approaches to Elicit Localized Immunity for Protection. Front. Cell Infect. Microbiol..

[137] Sugauchi F., Wang R.Y., Qiu Q., Jin B., Alter H.J., Shih J.W. (2006). Vigorous hepatitis C virus-specific CD4+ and CD8+ T cell responses induced by protein immunization in the presence of Montanide ISA720 plus synthetic oligodeoxynucleotides containing immunostimulatory cytosine-guanine dinucleotide motifs. J. Infect. Dis..

[138] Donnison T., von Delft A., Brown A., Swadling L., Hutchings C., Hanke T., Chinnakannan S., Barnes E. (2020). Viral. vectored hepatitis C virus vaccines generate pan-genotypic T cell responses to conserved subdominant epitopes. Vaccine.

[139] Gummow J., Li Y., Yu W., Garrod T., Wijesundara D., Brennan A.J., Mullick R., Voskoboinik I., Grubor-Bauk B., Gowans E.J. (2015). A Multiantigenic DNA Vaccine That Induces Broad Hepatitis C Virus-Specific T-Cell Responses in Mice. J. Virol..

[140] Gummow J., Masavuli M.G., Mekonnen Z.A., Li Y., Wijesundara D.K., Shrestha A.C., Voskoboinik I., Gowans E.J., Grubor-Bauk B. (2020). Safety Profile of a Multi-Antigenic DNA Vaccine Against Hepatitis C Virus. Vaccines.

[141] Wijesundara D.K., Gummow J., Li Y., Yu W., Quah B.J., Ranasinghe C., Torresi J., Gowans E.J., Grubor-Bauk B. (2018). Induction of Genotype Cross-Reactive, Hepatitis C Virus-Specific, Cell-Mediated Immunity in DNA-Vaccinated Mice. J. Virol..

[142] Gomez C.E., Perdiguero B., Cepeda M.V., Mingorance L., Garcia-Arriaza J., Vandermeeren A., Sorzano C.O., Esteban M. (2013). High, broad, polyfunctional, and durable T cell immune responses induced in mice by a novel hepatitis C virus (HCV) vaccine candidate (MVA-HCV) based on modified vaccinia virus Ankara expressing the nearly full-length HCV genome. J. Virol..

[143] Koutsoumpli G., Ip P.P., Schepel I., Hoogeboom B.N., Boerma A., Daemen T. (2019). Alphavirus-based hepatitis C virus therapeutic vaccines: Can universal helper epitopes enhance HCV-specific cytotoxic T lymphocyte responses?. Ther. Adv. Vaccines Immunother.

[144] Page K., Melia M.T., Veenhuis R.T., Winter M., Rousseau K.E., Massaccesi G., Osburn W.O., Forman M., Thomas E., Thornton K. (2021). Randomized Trial of a Vaccine Regimen to Prevent Chronic HCV Infection. N. Engl. J. Med..

[145] Christiansen D., Earnest-Silveira L., Grubor-Bauk B., Wijesundara D.K., Boo I., Ramsland P.A., Vincan E., Drummer H.E., Gowans E.J., Torresi J. (2019). Pre-clinical evaluation of a quadrivalent HCV VLP vaccine in pigs following microneedle delivery. Sci. Rep..

[146] Olivera S., Perez A., Falcon V., Urquiza D., Pichardo D., Martinez-Donato G. (2020). Protective cellular immune response against hepatitis C virus elicited by chimeric protein formulations in BALB/c mice. Arch. Virol..

[147] Filskov J., Andersen P., Agger E.M., Bukh J. (2019). HCV p7 as a novel vaccine-target inducing multifunctional CD4(+) and CD8(+) T-cells targeting liver cells expressing the Viral. antigen. Sci. Rep..

[148] Yusim K., Fischer W., Yoon H., Thurmond J., Fenimore P.W., Lauer G., Korber B., Kuiken C. (2010). Genotype 1 and global hepatitis C T-cell vaccines designed to optimize coverage of genetic diversity. J. Gen. Virol..

[149] Burke K.P., Munshaw S., Osburn W.O., Levine J., Liu L., Sidney J., Sette A., Ray S.C., Cox A.L. (2012). Immunogenicity and cross-reactivity of a representative ancestral sequence in hepatitis C virus infection. J. Immunol..

[150] Yusim K., Dilan R., Borducchi E., Stanley K., Giorgi E., Fischer W., Theiler J., Marcotrigiano J., Korber B., Barouch D.H. (2013). Hepatitis C genotype 1 mosaic vaccines are immunogenic in mice and induce stronger T-cell responses than natural strains. Clin. Vaccine Immunol..

[151] Fischer W., Perkins S., Theiler J., Bhattacharya T., Yusim K., Funkhouser R., Kuiken C., Haynes B., Letvin N.L., Walker B.D. (2007). Polyvalent vaccines for optimal coverage of potential T-cell epitopes in global HIV-1 variants. Nat. Med..

[152] Barouch D.H., Tomaka F.L., Wegmann F., Stieh D.J., Alter G., Robb M.L., Michael N.L., Peter L., Nkolola J.P., Borducchi E.N. (2018). Evaluation of a mosaic HIV-1 vaccine in a multicentre, randomised, double-blind, placebo-controlled, phase 1/2a clinical trial (APPROACH) and in rhesus monkeys (NHP 13-19). Lancet.

[153] Lee P.S., Zhu X., Yu W., Wilson I.A. (2015). Design and Structure of an Engineered Disulfide-Stabilized Influenza Virus Hemagglutinin Trimer. J. Virol..

[154] Yassine H.M., Boyington J.C., McTamney P.M., Wei C.J., Kanekiyo M., Kong W.P., Gallagher J.R., Wang L., Zhang Y., Joyce M.G. (2015). Hemagglutinin-stem nanoparticles generate heterosubtypic influenza protection. Nat. Med..

[155] Impagliazzo A., Milder F., Kuipers H., Wagner M.V., Zhu X., Hoffman R.M., van Meersbergen R., Huizingh J., Wanningen P., Verspuij J. (2015). A stable trimeric influenza hemagglutinin stem as a broadly protective immunogen. Science.

[156] Torrents de la Pena A., Julien J.P., de Taeye S.W., Garces F., Guttman M., Ozorowski G., Pritchard L.K., Behrens A.J., Go E.P., Burger J.A. (2017). Improving the Immunogenicity of Native-like HIV-1 Envelope Trimers by Hyperstabilization. Cell Rep..

[157] Henderson R., Edwards R.J., Mansouri K., Janowska K., Stalls V., Gobeil S.M.C., Kopp M., Li D., Parks R., Hsu A.L. (2020). Controlling the SARS-CoV-2 spike glycoprotein conformation. Nat. Struct. Mol. Biol..

[158] McCallum M., Walls A.C., Bowen J.E., Corti D., Veesler D. (2020). Structure-guided covalent stabilization of coronavirus spike glycoprotein trimers in the closed conformation. Nat. Struct. Mol. Biol..

[159] Rantalainen K., Berndsen Z.T., Antanasijevic A., Schiffner T., Zhang X., Lee W.H., Torres J.L., Zhang L., Irimia A., Copps J. (2020). HIV-1 Envelope and MPER Antibody Structures in Lipid Assemblies. Cell Rep..

[160] Lauer K.B., Borrow R., Blanchard T.J. (2017). Multivalent and Multipathogen Viral. Vector Vaccines. Clin. Vaccine Immunol..

[161] Keck Z.Y., Pierce B.G., Lau P., Lu J., Wang Y., Underwood A., Bull R.A., Prentoe J., Velazquez-Moctezuma R., Walker M.R. (2019). Broadly neutralizing antibodies from an individual that naturally cleared multiple hepatitis C virus infections uncover molecular determinants for E2 targeting and vaccine design. PLoS Pathog..

[162] Chen F., Nagy K., Chavez D., Willis S., McBride R., Giang E., Honda A., Bukh J., Ordoukhanian P., Zhu J. (2020). Antibody Responses to Immunization With HCV Envelope Glycoproteins as a Baseline for B-Cell-Based Vaccine Development. Gastroenterology.

[163] Chen F., Tzarum N., Lin X., Giang E., Velazquez-Moctezuma R., Augestad E.H., Nagy K., He L., Hernandez M., Fouch M.E. (2021). Functional convergence of a germline-encoded neutralizing antibody response in rhesus macaques immunized with HCV envelope glycoproteins. Immunity.

[164] Berggren K.A., Suzuki S., Ploss A. (2020). Animal Models Used in Hepatitis C Virus Research. Int. J. Mol. Sci..

[165] Billerbeck E., Wolfisberg R., Fahnoe U., Xiao J.W., Quirk C., Luna J.M., Cullen J.M., Hartlage A.S., Chiriboga L., Ghoshal K. (2017). Mouse models of acute and chronic hepacivirus infection. Science.

